# What Is Food Noise? A Conceptual Model of Food Cue Reactivity

**DOI:** 10.3390/nu15224809

**Published:** 2023-11-17

**Authors:** Daisuke Hayashi, Caitlyn Edwards, Jennifer A. Emond, Diane Gilbert-Diamond, Melissa Butt, Andrea Rigby, Travis D. Masterson

**Affiliations:** 1 Department of Nutritional Sciences, The Pennsylvania State University, University Park, PA 16801, USAtravis.d.masterson@psu.edu (T.D.M.); 2 Department of Biomedical Data Science, Geisel School of Medicine, Dartmouth College, Hanover, NH 03755, USA; 3 Department of Public Health Sciences, Penn State College of Medicine, Hershey, PA 17033, USA; 4 Penn State Health, Milton S. Hershey Medical Center, Hershey, PA 17033, USA

**Keywords:** craving, cue reactivity, eating behavior, food noise, GLP-1RAs

## Abstract

As GLP-1 receptor agonists, like semaglutide, emerge as effective treatments for weight management, anecdotal reports from patients and clinicians alike point to a reduction in what has been colloquially termed “food noise”, as patients report experiencing less rumination and obsessive preoccupation about food. In this narrative review, we discuss concepts used in studies to investigate human eating behavior that can help elucidate and define food noise, particularly food cue reactivity. We propose a conceptual model that summarizes the main factors that have been shown to determine the magnitude of the reactivity elicited by external and internal food cues and how these factors can affect short- and long-term behavioral and clinical outcomes. By integrating key research conducted in this field, the Cue–Influencer–Reactivity–Outcome (CIRO) model of food cue reactivity provides a framework that can be used in future research to design studies and interpret findings related to food noise and food cue reactivity.

## 1. Introduction

In the past decade, important advancements have been made in improving the medical treatment of obesity, including the more widespread use of bariatric surgery and obesity-related medications [1]. These changes have become particularly apparent with the widespread use of glucagon-like peptide-1 receptor agonists (GLP-1RAs). Two GLP-1RAs that were originally developed and approved for treating type 2 diabetes are now approved by the U.S. Food and Drug Administration (FDA) for weight management—namely, liraglutide and the more effective semaglutide [2,3]. For instance, in the STEP 1 clinical trial, which included 1961 adults with a body mass index above 27 kg/m^2^, semaglutide 2.4 mg in addition to a lifestyle intervention was associated with a mean change in body weight of −14.9% after 68 weeks [4], as well as a reduction in total and visceral fat mass and an increase in the proportion of lean body mass [5]. Other GLP-1RAs—like the dual and triple agonists tirzepatide and retratutide [6,7,8]—are currently being studied for the same application. This new generation of medications has shown substantial results for the treatment of obesity, leading to dose-dependent weight loss effects that surpass 20% of total body weight, accompanied by marked improvements in cardiometabolic health when paired with lifestyle interventions [1,9].

There are several theorized reasons for the success of GLP-1RAs in the treatment of obesity. For example, GLP-1RAs have been shown to activate GLP-1 receptors in numerous tissues and organs in the human body, three of which are believed to be of particular relevance for weight management: the gastrointestinal tract, the pancreas, and parts of the central nervous system [2]. In the gastrointestinal tract, GLP-1RAs can delay gastric emptying and glucose absorption, promoting greater satiety between meals [10]. In the pancreas, they promote the release of insulin, which, in turn, helps regulate blood glucose and appetite [11].

Although GLP-1 receptors are expressed in many regions of the brain [12,13], there are conflicting results regarding whether GLP-1RAs can consistently cross the blood–brain barrier and affect the central nervous system as a whole [14]. However, drugs like semaglutide have been shown to affect areas of the brain involved in the regulation of appetite and reward-seeking behaviors, including the hypothalamus, which can be exposed to semaglutide through tanycytes lining the ventricle wall that may help to regulate eating behaviors, thus contributing to an overall reduction in energy intake [15,16]. Similarly, in a recent study with rodent models, semaglutide reduced alcohol drinking, potentially by modulating GABA neurotransmission in the central amygdala and infralimbic cortex [17], suggesting that GLP-1RAs might also have an impact on addictive behaviors, which share many neural pathways with eating behaviors [18,19,20].

In addition to these mechanisms proposed by researchers as potential drivers of the weight loss effects of these drugs in clinical trials [4,21], patient-reported anecdotal evidence points to a construct that could be either the direct culmination of such aforementioned mechanisms or a separate effect of GLP-1RAs; patients have noted a reduction in what has colloquially been termed “food noise”. This suggests that there are likely psychological benefits to the use of these medications for the treatment of obesity, which may allow for additional intervention and treatment strategies to further enhance patient weight loss. In this narrative review, we will attempt to elucidate the construct of “food noise” as well as the methods available in the literature to assess it.

## 2. The Anecdotal Evidence on “Food Noise”

With the widespread use of GLP-1RAs and the positive results observed in real-life clinical settings [22], news articles have been documenting such anecdotal evidence from patients reporting that, after using GLP-1RAs (particularly semaglutide) for weight management, the “food noise” inside their heads has quieted. Such patients report previously experiencing constant and persistent thoughts about foods and eating that were difficult to suppress, to the point of feeling as if their lives revolved around food [23,24,25]. A few examples of anecdotal accounts of what patients call “food noise” are as follows: thinking about foods all the time (particularly highly palatable and energy-dense foods), feeling tempted to check food delivery applications multiple times a day, and thinking about the next meal they will consume when eating a meal [26,27]. This kind of rumination and obsessive preoccupation about food has been recently referred to as food-related intrusive thoughts (FRITs), which are believed to be experienced by people with and without clinically diagnosed eating disorders alike, particularly when struggling with their body weight or body image [28]. Such a phenomenon might pose a significant barrier to successful lifestyle changes, contributing to overeating and maladaptive eating behaviors, including emotional eating [29].

Although patient and clinician testimonies pointing to a reduction in FRITs seem to be a promising additional benefit that might help explain the effectiveness of GLP-1RAs on the treatment of obesity, there is still a need for systematically investigating this kind of effect and conceptualizing this construct, or collection of distinct constructs, that patients refer to as “food noise” so that scientists might be able to measure how it varies and to elucidate its role in weight management. While “food noise” may be a new concept to the public, we argue herein that researchers have been utilizing alternative terminology to describe an intimately related construct that can help explain “food noise”, namely, food cue reactivity.

## 3. Food Cue Reactivity: An Adaptive Characteristic That Can Lead to Maladaptive Eating Behaviors

Humans possess an extensive mesocorticolimbic circuitry that is exceptionally efficient at eliciting motivational responses upon exposure to food cues [30,31]. In plain language, our brains are experts at making us desire the foods and beverages we see, smell, and hear (e.g., the sound of bacon sizzling in a skillet). These motivational responses that arise from reactivity to food cues are consciously experienced as food-related cognitions and food cravings, defined as intense desires or urges to consume food. These responses can manifest regardless of physiological hunger levels and ultimately lead to increased food-seeking and consumption behaviors [30,32].

From an evolutionary perspective, such mechanisms have contributed to the survival of our species in times of scarcity, allowing humans to respond to opportunities to meet their nutritional needs whenever resources were available [33]. However, in most current food environments in industrialized countries, people are constantly presented with such opportunities to consume and are surrounded by food cues associated with highly palatable and energy-dense foods, which can contribute to overeating and obesity [34,35].

One example of such changes in the food environment that can contribute to constant exposure to food cues is the multiple media channels through which individuals are exposed to food advertisements. Digital and social media platforms have been specifically designed to be attention-grabbing and yield the highest possible reactivity to ultimately elicit intense food cravings, shape attitudes, and increase consumption [36]. Exposure to food advertisements on social media and video game streaming platforms [37,38] are relatively recent additions to the arsenal of means of communication employed by advertisers and can arguably extend the reach of food marketing and the number of food cues an individual may encounter in their physical and digital environments.

Individual differences in reactivity to food cues might help to partially explain why some people are more susceptible than others to overeating and developing obesity when living in similar environments. For example, heightened cue reactivity, which is often assessed as self-reported preoccupation with food or measured using changes in brain response to food cues, has been observed in overweight individuals [39] and those living with a binge-eating disorder [40]. These data seem to indicate that similar levels of exposure to food cues might lead to different levels of food noise experienced by individuals, which manifest as rumination and obsessive preoccupation with food (i.e., FRITs) and can lead to overeating and maladaptive eating behaviors. If the anecdotal evidence that “food noise” is dampened with the use of GLP-1RAs is confirmed through systematic investigation, it is possible that food cue reactivity can be modified with pharmacological therapy, preventing the occurrence of food noise. For the sake of a definition, food noise could be described as heightened and/or persistent manifestations of food cue reactivity, often leading to food-related intrusive thoughts and maladaptive eating behaviors (Table 1).

## 4. The Cue–Influencer–Reactivity–Outcome (CIRO) Model of Food Cue Reactivity

**Figure 1:**
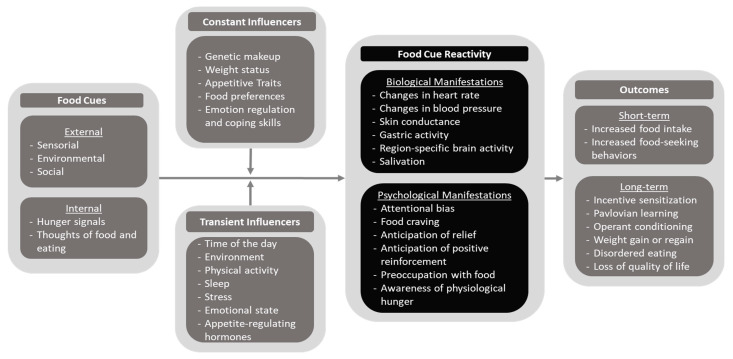
The CIRO model of food cue reactivity.

While many researchers have studied and described food cue reactivity, as documented in a literature review by Kanoski and Boutelle [40], there is currently no unifying model that maps out the evidence regarding the range of contributing factors and how those factors are manifested. Therefore, we sought to assess and summarize the current state of the literature and concepts related to food cue reactivity, including its determinants and manifestations within and beyond the context of overweight and maladaptive eating behaviors. This process resulted in the development of the Cue–Influencer–Reactivity–Outcome (CIRO) model of food cue reactivity (Figure 1).

In this model, the presence of food cues elicits varying degrees of food cue reactivity, which is influenced by constant and transient influencers and, when heightened, ultimately contributes to overeating and weight gain [40]. Most importantly, this model highlights that cue reactivity is likely to be influenced by many factors, some of which are constantly present and others that are continually changing, which can help to explain the dynamic nature of food cue reactivity. To account for this high level of potential variability, our conceptual model considers two categories of potential cues: external food cues, which arise from the physical and social environment (e.g., seeing and smelling palatable foods), and internal food cues, such as those that might arise from homeostatic hunger signals (e.g., noticing that your stomach is growling) and thoughts about food and eating [41].

In addition to the food cue itself, we consider factors that may modify the strength of the responses elicited in response to it, including constant (e.g., genetics) and transient (e.g., time of day) factors. Constant factors such as certain appetitive traits (e.g., those assessed by the Adult and Children Eating Behavior Questionnaires) [42,43], a person’s genetic makeup, usual food preferences, and weight status are all associated with different levels of reactivity to food cues [44,45,46,47,48,49]. We also considered skills related to emotion regulation and coping as constant influencers, since previous research has shown that employing strategies such as cognitive reappraisal can help manage neural reactivity to food cues and self-reported cravings for energy-dense foods [50].

Similarly, transient factors and states, such as the time of day, surrounding environment, sleep duration, level of psychological stress, emotional state, physical activity, and levels of appetite-regulating hormones (e.g., circulating levels of leptin, ghrelin, PYY, endogenous GLP-1, and GLP-1RAs), have been associated with varying levels of food cue reactivity [46,51,52,53,54,55,56,57,58,59,60,61].

## 5. Methods for Assessing Food Cue Reactivity

After considering these modifying factors, we then mapped to our model how food cue reactivity manifests and how it can be assessed, so that researchers interested in studying food cue reactivity and food noise are presented with an array of options to choose from when measuring such constructs. We, therefore, compiled examples of methods that have been previously employed in the broader literature to assess food cue reactivity in humans. We then divided these measures into two groups of manifestations based on how they are typically measured in these studies.

## 6. Biological Manifestations of Food Cue Reactivity

The first group of manifestations of food cue reactivity is biological (or physiological) manifestations, which include a range of measurable cephalic phase responses to food cues, including changes in heart rate, blood pressure, skin conductance, gastric activity, and salivation [41,53,62]. Other important biological manifestations of food cue reactivity that have been extensively studied are changes in brain activity that can be measured with functional magnetic resonance imaging (fMRI) [48,63], electroencephalography (EEG), functional near-infrared spectroscopy (fNIRS), and magnetoencephalography (MEG) [49]. The most widely employed of such methods in the context of food cue reactivity is fMRI.

With fMRI, researchers can measure brain responses to food cues in laboratory settings using either an exploratory approach (with whole-brain fMRI) or focusing on brain regions associated with different aspects of eating behavior by conducting region-of-interest (ROI) analyses [64]. The use of fMRI to assess food cue reactivity presents several strengths, such as providing an objective measure of brain activity and allowing for investigating the responses of different regions of interest to food cues, which can help understand how distinct aspects of food cue-related brain responses might change due to individual differences and in response to treatments [65]. The downside of this approach is the high cost of equipment and trained personnel and the low ecological validity, as assessing responses to food cues in a laboratory setting while wearing a brain scanner is contextually very different from free-living conditions [66]. Despite the measurement of such biological manifestations of food cue reactivity being inherently limited in ecological validity and not feasible when considering conducting a dense repeated measure field study, they could be of great value to elucidating changes in food cue reactivity before and after different treatments in clinical settings, such as in patients being treated with GLP-1RAs.

## 7. Psychological Manifestations of Food Cue Reactivity

The second category of methods we have compiled pertains to psychological manifestations of food cue reactivity, which includes changes in attentional bias (most often measured with eye-tracking and reaction-time-based paradigms and the emotional Stroop task) [59,67,68] and questionnaires that measure trait- and state-level constructs that can influence eating behaviors [69]. One example of a questionnaire that can be used to assess psychological manifestations of food cue reactivity is the Food Cue Responsivity Scale [70], which was recently developed by Sim and colleagues by combining items from pre-existing scales aimed at assessing reactivity to distinct types of food cues across two subdomains: uncontrolled eating behavior and cognitive rumination.

Another useful tool for studying psychological manifestations of food cue reactivity is a set of questionnaires widely employed in eating behavior research that allow for distinguishing between reactivity to food cues as a trait and a state—the state and trait versions of the Food Craving Questionnaire [69,71]. This distinction could be particularly useful in studies aiming at evaluating reactivity to food cues in a dynamic manner and allowing for identifying within-subject variation after repeated measures. There are also adaptations of the state and trait versions of the Food Craving Questionnaire focusing on general food cravings rather than craving specific foods, developed by Nijs et al. [72]. It is worth noting that, although the name of these questionnaires might suggest they are limited to assessing food craving, their scores are computed under five subdomains, which assess much broader manifestations of food cue reactivity. Table 2 provides an example question, which is rated on a scale ranging from 1 (strongly disagree) to 5 (strongly agree), from each of the five subdomains of the General Food Cravings Questionnaire–State (G-FCQ-S).

Yet another example of a measure that can be employed to assess psychological manifestations of food cue reactivity is the Visual Analog Scale (VAS). VAS can be easily adapted, giving researchers the ability to utilize or adapt different questions that might be related to food cue reactivity and eating behavior. VAS can be used to rate an individual’s thoughts and feelings by marking a point in a straight line or slider with two opposite answers as anchors on its extreme sides. One example of this approach is the following set of six questions [73], employed by Masterson et al. [51], to evaluate participants’ perceptions of hunger and preoccupation with food at different times of the day: (i) How hungry do you feel?; (ii) How full do you feel?; (iii) How strong is your desire to eat?; (iv) How much do you think you could eat now?; (v) What is your urge to eat?; and (vi) What is your preoccupation with thoughts of food? In this example, Masterson et al. found that preoccupation with food was higher in the evening compared to the morning, although hunger levels were similar.

## 8. Outcomes of Increased Food Cue Reactivity

In the final portion of the model, we classified the effects of increased food cue reactivity into short-term and long-term outcomes. The former represents behavioral responses elicited immediately or shortly after acute exposure to food cues, including increased food-seeking behaviors and food intake. Long-term outcomes, on the other hand, represent the results of repeated instances of exposure to food cues accompanied by heightened food cue reactivity and behavioral outcomes over prolonged periods, which can lead to the strengthening of the effectiveness of food cues on eliciting overeating through learning processes, including incentive sensitization [74,75], Pavlovian conditioning (i.e., a direct association between food cues and food intake), and operant conditioning (i.e., a reinforcement of food-seeking behaviors due to the rewarding nature of palatable foods) [40]. On top of these learning processes, increased food cue reactivity is associated, over time, with important health-related outcomes, including weight gain or regain [50,65,76], disordered eating behaviors and eating disorders [77,78,79], and, consequently, loss of quality of life [45,80].

In this paper, we used the example of how GLP-1RAs can mimic the appetite-regulating hormone GLP-1 and, thus, might influence food cue reactivity as one of its potential mechanisms that can help explain its remarkable results in weight management. Therefore, the anecdotal evidence from patients reporting the “silencing” of the “food noise” that constantly occupied their minds before obesity treatment using GLP-1RAs might represent a reduction in extreme forms of food cue reactivity, in which the magnitude of the food cue reactivity generated by exposure to food cues and their persistent nature is such that individuals struggle with food-related intrusive thoughts—FRITs. This reduction, in turn, might help explain the reduction in overeating and the positive clinical outcomes experienced by individuals undergoing obesity treatment with GLP-1RAs.

## 9. GLP-1RAs and Their Possible Role in Managing Behavioral Addictions and Substance Use Disorders: Additional Insights from the CIRO Model of Food Cue Reactivity

The CIRO model of food cue reactivity includes multiple manifestations of food cue reactivity under one construct (“R”) while acknowledging that the degree of these manifestations will vary between individuals and within individuals across time. Specifically, individual and environmental influencers that might be either constant or transient (“I”) modify the degree of food cue reactivity, and manifestations will be amplified by positive feedback after food consumption (“O”). “Food noise” thus reflects an advanced stage of food cue reactivity marked by a heightened and/or persistent preoccupation with food, a stage of reactivity that can lead to the occurrence of FRITs and have a negative impact on daily life (Table 1). That progression from typical to disordered food cue responsivity mirrors the development of preoccupation and disordered thinking in other behavioral addictions and substance use disorders [81]. Anecdotal evidence supports that treatment with semaglutide is related to reductions in substance use or compulsive behaviors other than eating (e.g., alcohol use, smoking, compulsive shopping) [82,83], suggesting that this drug may dampen maladaptive “noise” related to other rewarding behaviors. Animal models support that GLP-1RA treatment causally reduces alcohol intake, and GLP-1RAs may dampen the release of dopamine in the nucleus accumbens in response to alcohol intake as well as increase the breakdown of dopamine [17,84,85,86]. Alterations in dopaminergic pathways in the brain likely contribute to substance misuse [87,88] and play a role in compulsive behaviors [89]. Thus, it is possible that, in addition to acting upon pathways typically implicated in appetite regulation, GLP-1RAs dampen food noise by disrupting the development and reinforcement of disordered thought processes about food by acting on reward pathways in the brain.

## 10. Research and Public Health Implications

Based on the literature included in this narrative review, we theorize that the health-related outcomes of heightened and persistent food cue reactivity (i.e., “food noise”) portrayed in the CIRO model of food cue reactivity can be modified by intervening in the first two components of our model: a) people’s exposure to food cues, by modifying food environments, and b) factors that can influence food cue reactivity. While some of the factors that influence food cue reactivity are not currently modifiable in humans, such as their genetic makeup, others can be managed through medical and behavioral treatments. A combination of strategies is most likely to yield clinically significant benefits by addressing multiple influencers as well as the level of exposure to external food cues.

Furthermore, despite the compelling evidence of the benefits of GLP-1RAs as adjunct treatments to intensive behavioral therapy in weight management and their currently anecdotal effect on managing food noise, these drugs should not be regarded as standalone treatments, and care should be exercised when prescribing such pharmacological treatments and monitoring patients receiving them. As discussed by Powell et al. [90], semaglutide may display suboptimal results in real-world settings when used in isolation without the use of interventions to promote lifestyle changes, such as nutrition and psychological counseling, and adequate support to increase levels of physical activity. Additionally, concerns regarding a possible link between semaglutide use and depressive symptoms and suicidal ideation have recently emerged through case reports [91]. Despite the lack of statistically significant evidence for such association in clinical trials conducted so far [4,92], it is worth noting that the main trials for which data have been published (STEP 1 through STEP 5, STEP 8, and STEP TEENS) did not include participants who reported having symptoms of major depressive disorder within 2 years before screening, which limits their ability to assess psychiatric adverse events in people with a recent history of depression [3,4,93,94,95,96,97]. The European Medicines Agency (EMA) is currently reviewing data on about 150 reports of possible cases of suicidal thoughts and self-injury from patients treated with GLP-1RAs [98]. Although there is no evidence that these events have been directly caused by GLP-1RAs, these concerns reinforce the need for careful consideration and close monitoring of patients by obesity medicine teams, particularly for those with a history of major depressive disorder and suicidal ideation.

In order to maximize public health benefits, individuals, clinicians, public administrators, and policymakers should work together to address the biological, behavioral, social, and environmental determinants of health that lead to the development and maintenance of obesity by employing a socioecological approach to health promotion [99,100,101]. For example, it might be necessary to adopt multilevel, multicomponent strategies that combine individual-level interventions such as pharmacological and behavioral treatments that can help address influencers of food cue reactivity with community-level interventions and public policies that improve our food environments and regulate mass exposure to external food cues through media and advertisement, which are likely contributing to experiences of food noise [102]. Another essential goal should be improving equity in access to both medical and behavioral treatments through increased availability and insurance coverage, given that there are significant socioeconomic determinants of obesity and the gap is likely to become wider with the emergence of new treatments that are prohibitively costly for people of lower income [103,104].

As well as developing policies that can limit exposure to food cues and improving access to treatments that can modify influencers of food cue reactivity, it might also be necessary to address motivational barriers regarding seeking such treatments. At the same time, as our environments shift toward favoring overeating and obesity due to constant exposure to food cues, the societal standards in industrialized countries also tend to promote widespread weight stigma and body shame [105], which, in addition to leading to stress and favoring emotional eating [106], can contribute to the avoidance of treatment-seeking behaviors due to fear of experiencing weight discrimination in healthcare settings [107]. This fear of discrimination and embarrassment due to one’s body weight and/or eating behaviors is commonly experienced by people suffering from binge-eating disorder, for whom such fear manifests as attempts to hide one’s behavior from others and eat in isolation [108]. Such attempts can make maladaptive eating behaviors harder to identify and treat. The same might occur with experiences of food noise, which can remain undisclosed by patients due to fear of judgment.

There are plenty of promising areas for future research on understanding and managing food cue reactivity. A few examples include investigating which of the determinants of food cue reactivity present in the literature have the greatest impact on human eating behavior and how clinically and demographically distinct populations are affected by them in different contexts. For instance, understanding the impact of emerging weight management drugs (e.g., GLP-1RAs, like semaglutide, and the newer dual and triple agonists tirzepatide and retratutide) [6,7,8] on food cue reactivity and systematically investigating their ability to halt the progression of food cue reactivity to food noise and what happens once patients stop using such medications is yet another promising area of research, as the anecdotal evidence from such effects promoted by GLP-1RAs adds yet another layer of complexity to the interplay between the psychosocial and biological influencers of food cue reactivity. Particularly, extending laboratory findings to ecologically valid contexts should also be a research priority, given that the way people experience food cue reactivity might differ in free-living conditions. Lastly, research should focus on improving current medical and behavioral treatments to manage food cue reactivity and food noise (e.g., developing and testing non-pharmacological treatments that could also contribute to reducing food noise, such as environmental enrichment and the use of rewarding activities other than eating) [109,110], as well as public policies that can yield significant clinical and public health impacts.

It is important to note that the CIRO model of food cue reactivity does not aim to work as an all-encompassing model that explains all the possible variables and relationships that influence how humans respond to food cues. Instead, the constructs and directions of the arrows displayed in the model are meant to provide a simplified visual representation of the current research describing how different measurable factors impact food cue reactivity. The actual relationships between variables are likely much more complex than those represented in the CIRO model. For example, certain influencers, like circulating levels of appetite-regulating hormones and time of the day, are likely to trigger internal food cues (e.g., feelings of hunger and thoughts about food and eating) on their own, in addition to influencing the magnitude of the food cue reactivity elicited by those cues, and can even influence each other (in this example, the time of the day can also affect the level of appetite-circulating hormones due to influences of circadian rhythm and eating patterns) [111]. Similarly, emotion regulation and coping skills are likely to also affect the degree to which food cue reactivity determines behavioral outcomes, as even individuals experiencing food-related intrusive thoughts might be less likely to engage in maladaptive eating behaviors when using the right coping strategies. Additionally, the distinction between constant and transient influencers can be blurred in situations where transient influencers that can increase food cue reactivity, such as stress, might become chronic—like that experienced within food insecure households and by individuals from minority groups who face day-to-day discrimination, both of which have been shown to experience increased levels of food cue reactivity compared to those living in food secure households and those who report lower levels of experienced discrimination [112,113]. This nuanced interplay of factors should be taken into consideration by researchers when designing studies and interpreting their findings, and future research could lead to updating the proposed model. The model may also be expanded in the future as new evidence points to additional factors that can influence food cue reactivity and food noise.

## 11. Conclusions

Despite the lack of systematic research on the phenomenon of “food noise”, there is growing anecdotal evidence that the use of GLP-1RAs, particularly semaglutide, might reduce “food noise”. We have defined “food noise” as “heightened and/or persistent manifestations of food cue reactivity, often leading to food-related intrusive thoughts and maladaptive eating behaviors”. Ultimately, this narrative review aimed to define “food noise” and discuss how it relates to the more widely studied construct of food cue reactivity. Moreover, based on the key literature on human eating behavior, we proposed a more unified conceptual model of food cue reactivity that can help researchers understand the factors that affect food cue reactivity and its outcomes on human eating behavior and health—the Cue–Influencer–Reactivity–Outcome (CIRO) model of food cue reactivity. This model provides a helpful scaffold for the study of food cue reactivity and food-related cognitions.

## Figures and Tables

**Table 1:** Definitions of terms and constructs mentioned in this paper.

Term	Definition
Food cue	External and internal conditioned stimuli that can elicit food-related responses, including sensorial (e.g., seeing and smelling food), environmental (e.g., walking by one’s favorite restaurant), and social (e.g., seeing people eating) cues, as well as internal hunger cues (e.g., one’s stomach growling).
Food cue reactivity	Conditioned responses to food cues, including physiological and psychological manifestations that favor food-seeking behaviors.
Food noise	Heightened and/or persistent manifestations of food cue reactivity, often leading to food-related intrusive thoughts and maladaptive eating behaviors.

**Table 2:** G-FCQ-S items [72].

Subscale Domain	Example Question
An intense desire to eat	I’m craving tasty food
Anticipation of relief from negative states	If I ate something, I wouldn’t feel so sluggish and lethargic
Craving as a physiological state	If I ate right now, my stomach wouldn’t feel as empty
Obsessive preoccupation with food	My desire to eat something tasty seems overpowering
Anticipation of positive reinforcement	If I were to eat what I’m desiring, I am sure my mood would improve

## Data Availability

Not applicable.
